# Effect of early preventive supplementation with calcium and phosphorus on metabolic bone disease in premature infants

**DOI:** 10.1186/s12887-024-04654-w

**Published:** 2024-03-08

**Authors:** Xuejing Xu, Hongfang Ma, Shuqi Cheng, Jiang Xue

**Affiliations:** 1 Department of Neonatology, The Second Hospital, Cheeloo College of Medicine, Shandong University, Jinan, 250033 China; 2 The Second Clinical Medical School of Shandong University, Jinan, 250033 China

**Keywords:** Premature, Metabolic bone disease, Phosphorus, Alkaline phosphatase, 25 hydroxyvitamin D

## Abstract

**Objective:**

The objective was to study the effect of early preventive calcium and phosphorus supplementation on metabolic bone disease in preterm infants.

**Methods:**

A retrospective analysis of 234 preterm infants with a gestational age < 32 weeks or birth weight < 1500 g who were hospitalized in the Neonatology Department of the Second Hospital of Shandong University from 01.2018 to 12.2020 was conducted. One hundred thirty-two premature infants hospitalized from 01.2018 to 06.2019 did not receive prophylactic calcium and phosphorus supplementation in the early postnatal period. These infants received calcium or phosphorus supplementation at the time of hypocalcaemia or hypophosphatemia diagnosis. One hundred two premature infants hospitalized from 07.2019 to 12.2020 received early preventive calcium and phosphorus supplementation after birth. The levels of serum calcium and phosphorus, alkaline phosphatase, 25-hydroxyvitamin D, calcitonin, and parathyroid hormone at different time points and growth indicators at six months of age were compared between the two groups of infants. The number of cases of metabolic bone disease and fracture between the two groups was compared.

**Results:**

1) A total of 12 infants (5.13%) among the 234 preterm infants were diagnosed with metabolic bone disease, including 2 (1.96%) in the prophylactic supplementation group and 10 (7.58%) in the nonprophylactic supplementation group. Fractures occurred in 3 premature infants (25.0%) with metabolic bone disease, all of whom were in the group that did not receive prophylactic supplementation. 2) There was no significant difference in serum calcium and calcitonin levels between the two groups. The levels of serum phosphorus and 25 hydroxyvitamin D in the prophylactic supplementation group were higher than those in the nonprophylactic supplementation group (*P* < 0.05). In comparison, alkaline phosphatase and parathyroid hormone levels were lower in the prophylactic supplementation group than in the nonprophylactic supplementation group (*P* < 0.05). Preterm infants in the prophylactic supplementation group had higher weight, length, head circumference, and bone density values than those in the nonprophylactic supplementation group (*P* < 0.05).

**Conclusion:**

Preventive supplementation with calcium and phosphorus after birth can effectively improve calcium and phosphorus metabolism, and reduce the incidence of metabolic bone disease and fractures in premature infants. This can be further publicized and used clinically.

**Supplementary Information:**

The online version contains supplementary material available at 10.1186/s12887-024-04654-w.

## Introduction

Metabolic bone disease of prematurity (MBDP) is defined as the reduction of bone mineral content in preterm infants due to an imbalance in calcium and phosphorus metabolism in the body, resulting in abnormal ossification. The essence of MBDP is that the bone mineral content in premature infants cannot meet the needs for normal skeletal growth and development, and MBDP can be accompanied by blood biochemical and imaging changes [1]. The maximum foetal mineral accretion (calcium and phosphorus) occurs in the last trimester of pregnancy. Based on the severity of prematurity, preterm infants are deprived of this source [2, 3], and when their catch-up growth begins, their mineral needs are much higher than those of term infants on a per unit body mass basis [2–4]. If preterm infants are not provided with additional mineral fortification, their skeletal growth will be abnormal, eventually causing abnormal bone growth, resorption, and mineral deposition. The clinical manifestations of MBDP are similar to those of rickets and fractures [1–3]. Gastrointestinal dysfunction, necrotizing enterocolitis (NEC), postgastrointestinal surgical conditions, and the use of diuretics (furosemide), steroids, or anticonvulsant drugs are also high-risk factors for MBDP [2, 3]. In preterm infants, metabolic bone disease (MBD) involves chronic bone metabolism abnormalities, the incidence of which is negatively correlated with gestational age and birth weight [2, 3]. The clinical presentation of MBD occurs after blood biochemical and skeletal radiographic changes, and the diagnosis of MBD mainly depends on bone metabolism indicators combined with imaging. For a blood phosphorus level < 1.8 mmol/L and an alkaline phosphatase (ALP) level > 900 U/L, the sensitivity and specificity for diagnosing bone mineralization deficiency are 100% and 70%, respectively. These indicators were selected as the diagnostic criteria for MBD in this study [5, 6].

To reduce the incidence of MBDP and fractures, early detection and early intervention or treatment are needed. Current studies recommend early parenteral nutrition supplementation with calcium and phosphorus preparations, vitamin D, or vitamin AD [7, 8]. Premature infants should receive vitamin D or vitamin AD supplementation as soon as possible after birth, but there is no consensus on when and how to provide calcium and phosphorus supplementation. This study retrospectively analysed premature infants who received preventive calcium and phosphorus supplementation at different periods to investigate the effects of early calcium and phosphorus supplementation on mineral metabolism, MBD occurrence, and fracture in preterm infants.

## Materials and methods

### Subjects and subgroups

This was a single-centre retrospective study. Chinese very-low-birth-weight (VLBW) infants with a gestational age < 32 weeks or a birth weight < 1500 g who were admitted to the neonatal intensive care unit (NICU) of the Second Hospital of Shandong University between 01.2018 to 12.2020 were selected for the study. Infants who were born in this hospital and immediately transferred to the NICU were included. The exclusion criteria were as follows: ① congenital, inherited metabolic diseases and severe congenital malformations; ② the diagnosis of NEC or treatment with intestinal surgery; and ③treatment abandonment or clinical death. Premature infants who were hospitalized from 07–2019–12–2020 and received intravenous prophylactic calcium and phosphorus supplementation in the early postnatal period were included in the prophylactic supplementation group. Preterm infants who were hospitalized from 01–2018–06–2019 who did not receive intravenous calcium and phosphorus supplementation in the early postnatal period and those who received calcium or phosphorus supplementation after the diagnosis of hypocalcaemia (a total calcium level < 1.8 mmol/L or an ionic calcium level < 0.8 mmol/L) or hypophosphatemia (a blood phosphorus level < 1.8 mmol/L) were included in the nonprophylactic supplementation group.

### Methods

#### Basic feeding practices

The feeding regimen was based on the 2015 Canadian Guidelines for Feeding Preterm Infants [9] and the Chinese Guidelines for Feeding Premature Infants [10]. The preterm infants in this study were given minimal enteral feedings as early as possible within 24 h of birth. According to the time and quantity of maternal milk production, the type of feeding (premature infant formula or breast milk) at the time of milk initiation was decided, and a combination of birth weight, gestational age, and feeding tolerance was used to determine the rate of milk addition. Breast milk was gradually fortified when premature infants were fed up to 50–80 ml/kg of their mothers' own milk per day. Vitamin D supplementation was performed through the addition of vitamin D, vitamin AD and/or enteral feeding (formula and/or fortified breast milk) in the first week after birth to ensure that the total dose of vitamin D was 800–1000 IU/d. The ideal concentration of serum 25(OH)D in infants is at least 20 ng/ml, and we regularly monitor and adjust the supplemental dose [11]. We commonly use Human milk fortifiers produced by Synutra. Every 100 ml of fortified breast milk can provide an additional 140 IU of vitamin D, 75 mg of calcium, and 47.5 mg of phosphorus. We commonly use preterm infant formula produced by Nestle and Synutra. Every 100 ml of preterm infant formula can provide an additional 38-128 IU of vitamin D, 80.35-102 mg of calcium, and 47.52-56 mg of phosphorus.

#### Calcium and phosphorus supplementation methods

Calcium and phosphorus supplementation regimens for all preterm infants at our centre were determined according to the American Institute of Medicine, the European Society for Pediatric Gastroenterology, Hepatology and Nutrition, and the Chinese Guidelines for Clinical Application of Neonatal Nutrition Support. For partial parenteral nutrition (PN) in the early postnatal period, 24–40 mg/kg of elemental calcium and 18–30 mg/kg of elemental phosphorus were provided per day, and the ratio of calcium to phosphorus (mass ratio) was 1–1.3:1. For complete parenteral nutrition, the target amount of elemental calcium was 65–100 mg/kg/d, the target amount of elemental phosphorus was 50–80 mg/kg/d, and the calcium-phosphorus ratio could reach 1.7:1. After full enteral feeding, the calcium intake was 100–160 mg/kg/d and the phosphorus intake was 60–90 mg/kg/d, with a calcium to phosphorus ratio of 1.6:1–1.8:1 [9, 10]. In the prophylactic supplementation group, calcium gluconate and sodium glycerophosphate were added to the intravenous nutritional solution from Days 2–3 after birth, and minerals were supplemented through intravenous combined fortified feeding (formula or fortified breast milk) after the initiation of enteral feeding. If hypocalcaemia or hypophosphatemia was diagnosed, calcium and phosphorus supplementation was provided through oral paediatric calcium carbonate granules, sodium fructose diphosphate, and/or intravenous administration. In the nonprophylactic supplementation group, mineral supplementation was provided through fortified milk after enteral feeding, and parenteral nutrition did not include preventive calcium and phosphorus supplementation. For infants diagnosed with hypocalcaemia and hypophosphatemia, oral and/or intravenous mineral supplementation was provided. After premature infants received full enteral feeding (135–160 ml/kg/d), parenteral nutrition was discontinued.

All subjects in this study (gestational age < 32 weeks or birth weight < 1500 g) received intensive feeding and/or calcium and phosphorus supplements to ensure adequate mineral intake. During MBDP therapy (approximately 6–8 weeks), calcium and phosphorus supplementation was gradually decreased within 2–4 weeks after the biochemical indicators had improved and imaging showed signs of fracture healing or increased bone mass. Calcium-phosphorus therapy was discontinued when the blood phosphorus level returned to normal and the serum ALP level was < 500 U/L with a decreasing trend. Since all subjects in this study had high-risk factors for MBDP, they were all discharged from the hospital with continued intensive feeding until correction at full term or until regular clinical monitoring showed no evidence of combined MBDP. Bone metabolism and biochemical indicators of nutrition status were regularly assessed in high-risk infants and those diagnosed with MBDP who had already been discharged. When abnormalities were found, calcium and phosphorus supplementation was actively provided based on intensive feeding, combined with passive exercise, etc. If there were no abnormalities, regular monitoring was continued. A monitoring plan for postdischarge follow-up was developed based on the risk and severity of MBD high-risk factors. The time/frequency of monitoring were as follows: at discharge; every two weeks after discharge until postnatal correction at 1 month of age; monthly from 1–6 months of corrective age; every two months from 7–12 months of rectification age; every three months from 13–24 months of corrective age; and every six months after 24 months of postnatal correction.

#### Specimen collection and testing indices

Serum calcium, phosphorus, and ALP assays were collected from the 234 preterm infants at different time points (12 h, 15 days, 30 days, 45 days, 60 days, and 90 days). 25(OH)D assays were performed for all preterm infants at 15 days postnatally. Calcitonin (CT) and intact parathyroid hormone (PTH) levels were measured in some preterm infants (*n* = 54) at 30 days postnatally. Preterm infants with fewer than 30 days of hospitalization were followed up to measure the above indices. For newborns diagnosed with MBD or those at high risk for MBD, calcium and phosphorus supplementation and gentle care were provided. In the 66 infants who were diagnosed with MBD or at high risk of MBD (8 infants in the prophylactic supplementation group and 58 infants in the nonprophylactic supplementation group), the bone mineral density of the middle section of femur was examined by dual energy X-ray absorptiometry (DEXA, HOLOGIC Discovery Wi), and the speed of sound in the middle tibia was examined by HKANGYU quantitative ultrasound (QUS) Analyzer (HL-3302C) at six months of age to assess skeletal development.

#### Statistical analysis

SPSS 22.0 statistical software was used for data analysis. Nonnormally distributed variables are expressed as the median (M) (P_25_-P_75_), and the rank sum test was used for comparisons between groups. Count data are reported as the number of cases (percentages), and the chi-square test was used to compare groups. Measurement data are expressed as the mean ± standard deviation (mean ± SD), and intergroup comparisons were conducted using one-way ANOVA. The SNK method was used for pairwise comparisons between means. Comparisons of temporal trends for each group were performed using ANOVA with repeated measures data. A *P* value less than 0.05 was considered statistically significant.

### Diagnostic criteria

MBD was diagnosed when the ALP level was > 900 U/L and the blood phosphorus level was < 1.8 mmol/L [5, 6], and a high risk of MBD or intervention to prevent the occurrence of MBD was considered when the ALP level was > 500 U/L while ≤ 900 U/L and the blood phosphorus level was < 1.8 mmol/L [12].

## Results

### General information

**Fig. 1:**
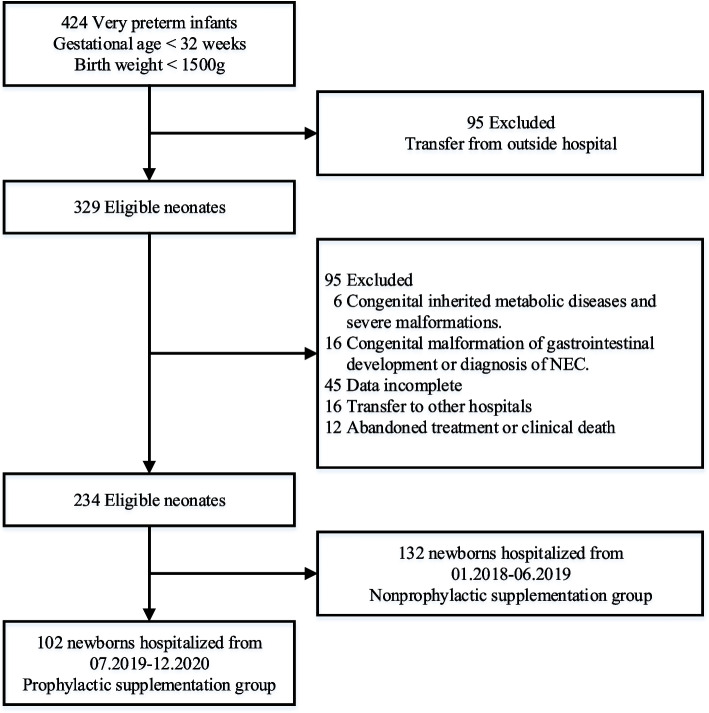
Flowchart for the current retrospective study

A total of 424 infants with a gestational age < 32 weeks or a birth weight < 1500 g were treated in the NICU of the Second Hospital of Shandong University during the study period, of whom 329 were eligible for the study and 95 were subsequently excluded according to the exclusion criteria, resulting in a total of 234 infants being included in the survey (Fig. 1). In this retrospective analysis, there were 102 newborns in the prophylactic supplementation group and 132 newborns in the nonpreventive supplementation group; there were 126 males and 108 females, with a median gestational age of 29.43 (27.43–30.71) weeks and a median birth weight of 1100 (900–1350) g (Table 1).

**Table 1:** Comparison of clinical data between the two groups

Projects	Nonprophylactic supplementation group	Prophylactic supplementation group	Statistics	*P*-value
Gestational age[M(P_25_ ~ P_75_)]/weeks	29.14(27.43 ~ 30.86)	29.57(27.86 ~ 31.14)	Z = 0.23	0.714
Birth weight[M(P_25_ ~ P_75_)]/g	1270(985.0 ~ 1552.0)	1300(1013.0 ~ 1585.0)	Z = 0.34	0.632
Gender(Male/Female, n)	71/61	55/47	*χ*^2^ = 0.31	0.787
MBD[n(%)]	10(7.58)	2(1.96)	*χ*^2^ = 5.53	0.023
High risk of MBD[n(%)]	48(36.36)	6(5.88)	*χ*^2^ = 9.47	0.016

**Fig. 2:**
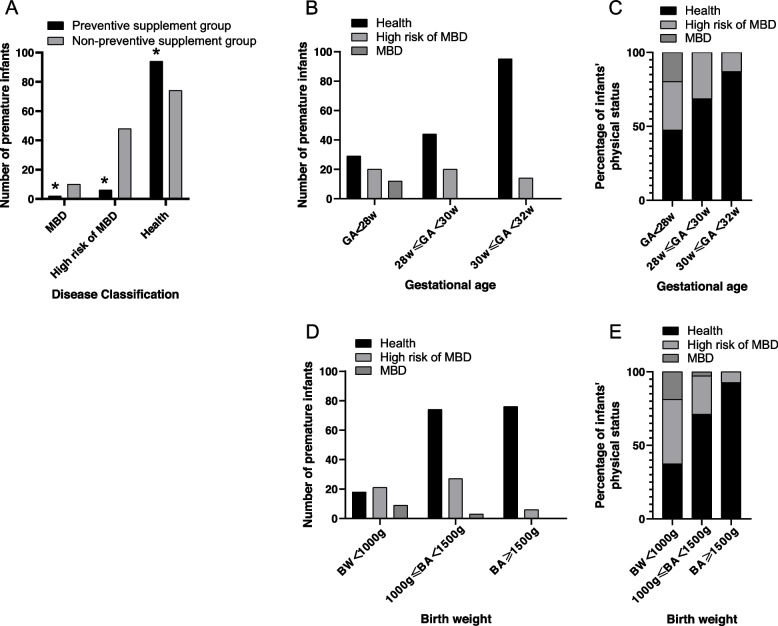
Distribution of metabolic bone disease and high risk of metabolic bone disease in the two groups of preterm infants (A. Compared to the nonprophylactic supplementation group, *P < 0.05)

In this retrospective study, 66 preterm infants were diagnosed with MBD or classified as being at high risk of MBD, with a total of 8/102 infants (2 with MBD, 6 at risk of MBD) in the prophylactic supplementation group and 58/132 infants (10 with MBD, 48 at risk of MBD) with MBD or at high risk of MBD in the nonprophylactic supplementation group. All premature infants with MBD had a gestational age < 28 weeks (*n* = 58, 20.69%), and 9 had an extremely low birth weight (birth weight < 1000 g, *n* = 46, 19.57%). The prevalence of a high risk of MBD in infants with gestational age < 28 weeks, between 28 weeks and ≤ 30 weeks, and between 30 weeks and ≤ 32 weeks was 30.39%, 28.44%, and 11.64%, respectively (Fig. 2). There were 3 cases of femur fracture in infants with MBD in the group that did not receive prophylactic supplementation, all of whom had a gestational age < 28 weeks and a birth weight ≤ 1000 g. Fractures were diagnosed in these infants on Days 53, 56, and 90, with an ALP level > 900 U/L, a phosphorus level < 1.6 mmol/L, and a calcium level > 1.8 mmol/L at the time of detection (Table 2).

**Table 2:** Fracture cases

Serial number	Gestational age(w)	Birth weight(g)	Days of hospitalization	Days of diagnosed	Fracture sites	Alkaline phosphatase (U/L)	Serum phosphorus(mmol/L)	Serum calcium (mmol/L)
1	27 + 5	1000	102	53	Femur	989	1.57	2.27
2	27 + 5	990	73	90	Femur	929	1.19	2.34
3	27 + 1	820	65	56	Femur	1009	0.96	2.67

### Comparison of biochemical parameters

#### Serum calcium and phosphorus levels

**Fig. 3:**
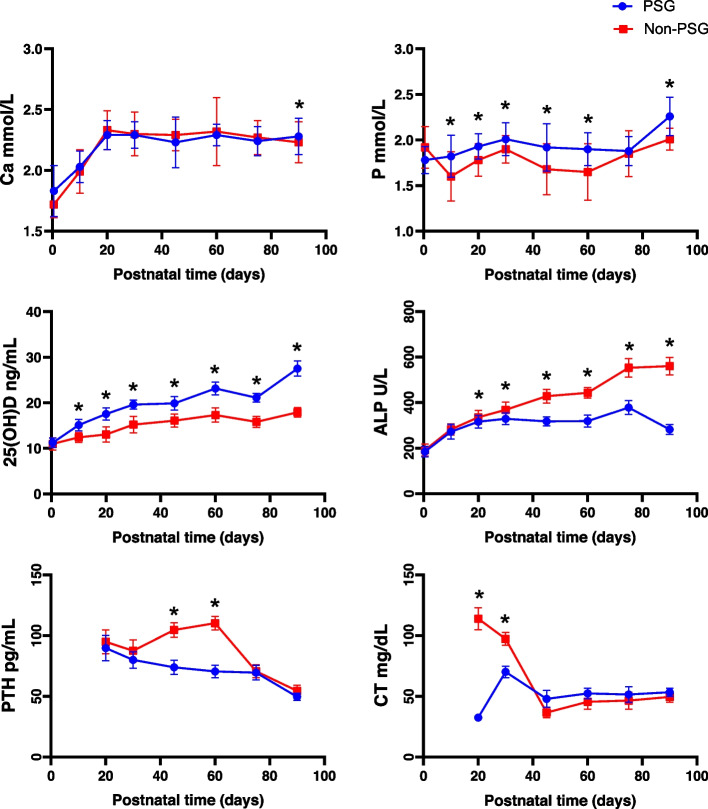
Comparison of serum biochemical indices between the two groups of preterm infants (mean ± SD. Comparison between groups: Compared to the nonprophylactic supplementation group during the same period, *P < 0.05)

The serum phosphorus levels in the prophylactic supplementation group were higher than those in the nonprophylactic supplementation group (*P* < 0.05). There was no significant difference in blood calcium levels between the two groups (Fig. 3).

#### Alkaline phosphatase levels

The serum ALP level increased gradually over time in both groups, and the ALP level in the prophylactic supplementation group was lower than that in the nonprophylactic supplementation group, and the differences were statistically significant (Fig. 3). The ALP level in the nonprophylactic supplementation group reached its highest value of 560.66 ± 38.65 U/L at 90 days.

#### Serum 25(OH)D levels

The serum 25(OH)D level increased gradually over time in both groups, and the 25(OH)D level in the prophylactic supplementation group was higher than that in the nonprophylactic supplementation group. These differences were statistically significant (*P* < 0.05).

#### Serum calcitonin and parathyroid hormone levels

CT and PTH tests were performed in 54 infants (25 infants in the prophylactic supplementation group and 29 infants in the nonprophylactic supplementation group); there was no significant difference in the CT level between the two groups. The serum PTH level in the preventive supplementation group was lower than that in the nonpreventive supplementation group, with statistically significant differences at postnatal days 45 and 60 (*P* < 0.05, Fig. 3).

### Comparison of growth indices

Growth indicators were assessed at six months postnatally in the 66 infants who were diagnosed with MBD or at high risk of MBD. The bone density, weight, and length of the prophylactic supplementation group were higher than those of the nonprophylactic supplementation group. These differences were statistically significant (*P *< 0.05, Fig. S1). All infants with fractures showed increased bone density on DEXA and good fracture end alignment, disappearing fracture lines, and healed fracture ends on X-rays at six months postnatally.

## Discussion

MBDP refers to a group of skeletal disorders characterized by reduced bone mineral content and incomplete mineralization of bone-like material due to insufficient calcium, phosphorus, and organic protein matrix or bone metabolism disorders in premature infants. The essence of MBD is that the bone mineral content in infants cannot meet the needs for normal bone growth and development, which can be accompanied by blood biochemical and imaging changes. MBDP has adverse effects on the health of infants. Recently, MBDP has been shown to be accompanied by ectopic growth retardation, ventilator dependence, and even fractures; in the long term, it may lead to short stature, rickets, osteoporosis, and so on [3, 13, 14]. Premature infants are prone to MBD due to insufficient calcium and phosphorus reserves, underdeveloped renal function, inadequate postnatal mineral supplementation, and delayed enteral feeding—the younger an infant's gestational age and the lower their birth weight is, the greater their risk of MBD. Therefore, early supplementation of mineral elements for preterm infants is significant [15, 16]. This study retrospectively investigated the clinical significance of early intravenous calcium and phosphorus supplementation on mineral metabolism and the occurrence of MBD in preterm infants.

A retrospective multicentre study in China found that the incidence of MBD in very-low-birth-weight (VLBW) and extremely low-birth-weight (ELBW) infants was 19.5% and 38.5%, respectively, while the incidence of MBD in infants with a gestational age < 32 weeks and < 28 weeks was 21.7% and 45.5%, respectively [17]. In this retrospective analysis, the decrease in the incidence of MBD was related to the clinician's awareness of high-risk factors for MBD (anticonvulsant use, steroid drug use, cholestasis, etc.) and aggressive enteral and parenteral nutrition support (such as fortified breast milk, supplementation with calcium and phosphorus and vitamin D). In addition, we found that aggressive calcium and phosphorus supplementation in the early postnatal period reduced the incidence of MBD and high-risk status.

Fractures and invasive ventilator dependence are the most common clinical features of MBD. MBD increases the risk of fractures, especially long bone and rib fractures. You et al. reported that the fracture rate was 11.7% in VLBW infants with MBD [18]. This study only detected fractures in 3 infants (2.27%) who did not receive prophylactic calcium and phosphorus supplementation. No fractures occurred in the preventive supplementation group, and the decrease in the incidence of MBD may be related to the improvement of infant care and mineral supplementation in recent years. Respiratory failure and withdrawal difficulties are seen in infants with MBD, which may be related to decreased bone mineral content affecting chest wall compliance. In this study, the increased duration of invasive and noninvasive mechanical ventilation in 12 premature infants with MBD may have been related to poor chest wall compliance.

Ninety-nine percent of the body's calcium is stored as hydroxyapatite in bones; the remaining 1% circulates in the blood as a component of blood calcium in the form of protein-bound or ionized calcium. CT and PTH levels coregulate blood calcium levels. When blood calcium levels decrease, the body mobilizes bone calcium to maintain the blood calcium levels under the regulation of PTH. Blood calcium levels may be normal or high when calcium is deficient in the body, and decreased calcium levels occur only when the bone calcium reserve is depleted at the late stage of MBD. Therefore, blood calcium levels have no value for the early diagnosis of MBDP [6]. This retrospective analysis showed no statistically significant difference in blood calcium levels between the two groups, which may be related to the fact that calcium is a threshold substance in nutrition. The absorption rate is high when calcium intake is low and in a specific range, while it is low when calcium intake is high. Calcium absorption does not increase when calcium intake reaches the corresponding level, which may also be related to the maintenance of calcium homeostasis by calcium-regulating hormones [15]. In this study, there was no significant difference in blood phosphorus levels between the two groups 12 h after birth, probably since 80% of the phosphorus in the body exists in the skeleton in the form of hydroxyapatite, and the concentrations of serum-free calcium and phosphorus affect the formation of bone salts. A calcium-phosphorus product equal to 40 contributes to bone mineralization. This may also be related to the insensitivity of hormones regulating calcium-phosphorus metabolism in premature infants of the early postnatal period [19, 20]. Maria et al. showed that hypophosphatemia is the earliest marker of mineral disorders, occurring 7–14 days postnatally [3]. Current research indicated that early phosphorus supplementation through parenteral nutrition could prevent the occurrence of MBD in VLBW [8] and ELBW [21] infants. This study found a significant difference in blood phosphorus levels between the two groups at 15 days postpartum. Blood phosphorus levels in the preventive supplementation group were higher than those in the nonpreventive supplementation group, consistent with previous studies. In summary, it is recommended that active supplementation with phosphorus and appropriate calcium supplementation in combination with calcium-phosphorus products and calcium-regulated hormone levels be provided in the early postnatal period for preterm infants.

Compared with the nonpreventive supplementation group, in the preventive supplementation group, serum 25(OH)D levels were significantly higher, and the incidence of MBD and high-risk status was considerably lower, suggesting that 25(OH)D may be closely related to the occurrence of MBD. 25(OH)D is the main form of vitamin D transported in the blood. The active form of vitamin D is 25(OH)D3, which participates in the regulation of mineral homeostasis by regulating intestinal calcium and phosphorus reabsorption, promoting the release of bone calcium and phosphorus into the blood, mediating bone resorption and formation, and participating in bone metabolism [22]. 25(OH)D was used as a detection indicator for vitamin D due to its high content and long half-life. Vitamin D and vitamin AD supplementation was provided for all infants in this study. Nevertheless, the 25(OH)D levels in the prophylactic supplementation group were higher than those in the nonprophylactic supplementation group. It was hypothesized that calcium and phosphorus levels in vivo may negatively affect the expression of mineral-regulating hormones. This study found that premature infants with a lower gestational age and birth weight had lower levels of 25(OH)D and a higher risk of developing MBD and high-risk conditions. This may be due to insufficient prenatal vitamin D intake, inadequate enteral feeding, and the lack of postnatal mineral supplementation. Studies have shown that 25(OH)D3 inhibits the differentiation of osteoblasts in early stages and suppresses the formation of mineralized nodules by osteoblasts. When osteoblasts mature, 25(OH)D3 can promote their differentiation and increase mineral deposition, indirectly inhibiting bone resorption [22]. The vitamin D concentration in the preventive supplementation group was higher than that in the nonpreventive supplementation group, and an appropriate concentration of vitamin D promotes bone formation by regulating calcium and phosphorus metabolism; when a mineral deficiency or abnormal calcium or phosphorus production occurs, vitamin D can regulate osteoblast differentiation and promote mineral absorption into the blood. Therefore, calcium, phosphorus, and vitamin D should be supplemented preventatively after birth to prevent the occurrence of MBD or fractures.

ALP is a ubiquitous membrane-bound glycoprotein that can be secreted by bones and the liver, kidneys, and intestines. Its physiological function is mainly to hydrolyse phosphate and pyrophosphate during osteogenesis, facilitating the osteogenic process. Since 90% of ALP exists in bone and is not regulated by PTH, ALP can be an essential indicator of bone mineralization. ALP shows a slight physiological increase 2–3 weeks after birth and may continue to rise with insufficient mineral supply. Mihatsch et al. proposed that osteoblast feedback increased ALP secretion during calcium-phosphorus dystrophy [6, 23]. Cholestasis, infections, glucocorticoids, and deficiencies in micronutrients such as copper and zinc can affect ALP levels and interfere with clinical judgement. Backstrom et al. confirmed that the diagnostic sensitivity of MBD was 100% for an ALP level > 900 IU/L and a blood phosphorus level < 1.8 mmol/L based on DEXA [5], which was also the diagnostic criterion in this study. This retrospective analysis revealed that ALP levels in the prophylactic supplementation group were lower than those in the nonprophylactic supplementation group. The ALP level in the preventive supplementation group decreased at 75–90 days postnatally. ALP levels did not decrease at 90 days in the nonpreventive supplementation group, and the average ALP value exceeded 500 U/L. Figueras-Aloy et al. found that an ALP level > 500 IU/L was associated with low bone mineral density [12], indicating that the preterm infants with higher ALP levels had a high likelihood of developing MBD.

PTH elevates blood calcium by increasing osteoclast activity, releasing bone calcium into the blood, promoting renal tubular calcium reabsorption, and enabling vitamin D synthesis and transformation. Negative feedback of the plasma calcium concentration regulates the secretion of PTH [6, 15]. There are currently no uniform PTH criteria for neonates. Pettifor et al. proposed that a serum PTH level > 100 pg/ml in ELBW infants indicates the risk of MBD [24]. Moreira et al. found that the diagnostic sensitivity for severe MBD was 100% in preterm infants with a birth weight < 1250 g and a PTH level > 180 pg/ml was combined with a blood phosphorus level < 1.5 mmol/L at three postnatal weeks [25]. The present study showed that PTH levels in the prophylactic supplementation group were lower than those in the nonprophylactic supplementation group, suggesting that postnatal prophylactic mineral supplementation may provide feedback on PTH levels and reduce the occurrence of MBD. CT is a hormone secreted by thyroid C cells, whose biological function is to lower blood calcium and phosphorus levels, with a short duration of action, and the main target organ is bone. CT is the only hormone that can effectively reduce blood calcium levels. CT has positive feedback regulation with blood calcium levels, and its physiological function is opposite that of PTH [15]. This study found that the CT levels in the preventive supplementation group were higher than those in the nonpreventive supplementation group at 45 days postnatally. Nevertheless, there was no statistically significant difference between the two groups, which was probably related to the insignificant difference in blood calcium levels between the groups. The 2021 Expert consensus on the clinical management of metabolic bone disease of prematurity no longer recommends CT levels as a screening indicator for MBD [6].

In summary, compared with preterm infants of the same gestational age and birth weight who did not receive prophylactic supplementation with calcium and phosphorus in the postnatal period, preterm infants with prophylactic supplementation had higher 25(OH)D and blood phosphorus levels and lower ALP and PTH levels. With the extension of birth time, there were still significant differences in the above biochemical indices between the two groups, even after providing adequate calcium and phosphorus supplementation and enteral feeding. The incidence of MBD and high-risk conditions was lower in the prophylactic supplementation group than in the nonprophylactic supplementation group. Preventive mineral supplementation in the early postnatal period can effectively improve calcium and phosphorus metabolic status, reduce the occurrence of MBD and high-risk conditions, and promote increased bone density in preterm infants, which can be promoted and used in clinical practice. The dose and duration of prophylactic calcium and phosphorus supplementation in very early preterm infants need to be comprehensively considered in combination with calcium, phosphorus, and ALP levels, gestational age and birth weight, and maternal calcium and vitamin D supplementation [8, 21], which is also a topic for further research. In addition, this study has shortcomings, such as being a single-centre retrospective study, having data from a relatively small sample, and having a short follow-up period. In future studies, we will expand the sample size, broaden the research scope, and extend the follow-up period to study the effect of early-life prophylactic calcium and phosphorus supplementation on metabolic bone disease in preterm infants.

## Conclusion

Preventive supplementation with calcium and phosphorus after birth can effectively improve calcium and phosphorus metabolism, and reduce the incidence of metabolic bone disease and fractures in premature infants. This can be further publicized and used clinically.

### Supplementary Information

**Additional file 1: Fig S1.** Comparison of growth indicators between the two groups of infants at the age of 6 months (PSG: prophylactic supplementation group, mean ± SD compared with the nonprophylactic supplementation group (non-PSG), *P < 0.05).

## Data Availability

The datasets used and/or analyzed during the current study are available from the corresponding author on reasonable request.

## Abbreviations

MBDP: Metabolic bone disease of prematurity
MBD: Metabolic bone disease
ALP: Alkaline phosphatase
NICU: Neonatal Intensive Care Unit
NEC: Necrotizing enterocolitis
25(OH)D: 25-Hydroxyvitamin D
CT: Calcitonin
PTH: Parathyroid hormone
SPSS: Statistical Packages for the Social Sciences
ANOVA: Analysis of Variance
VLBW: Very-low-birth-weight
ELBW: Extremely low-birth-weight
