# Supplementary material for: Effect of early preventive supplementation with calcium and phosphorus on metabolic bone disease in premature infants
Source: BMC Pediatr. 2024 Mar 8;24:171. doi: 10.1186/s12887-024-04654-w (PMC10921623; doi:10.1186/s12887-024-04654-w)
Supplement: Supplementary file 1 — Additional file 1: Fig S1. Comparison of growth indicators between the two groups of infants at the age of 6 months (PSG: prophylactic supplementation group, mean ± SD compared with the nonprophylactic supplementation group (non-PSG), *P < 0.05). [file 12887_2024_4654_MOESM1_ESM.pdf]

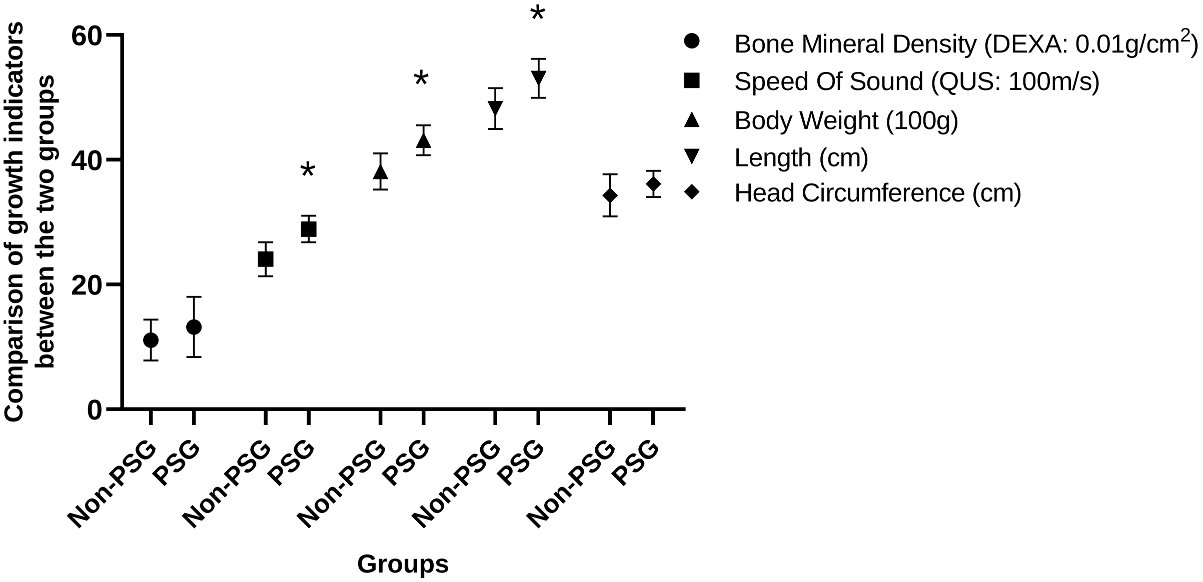

Fig S1 Comparison of growth indicators between the two groups of infants at the age of 6 months (PSG: prophylactic supplementation group, mean±SD compared with the nonprophylactic supplementation group (non-PSG), *P<0.05).
